# Case Report: Ethanol ablation of the Marshall vein as the first step for left atrial tachycardia ablation

**DOI:** 10.3389/fcvm.2024.1431736

**Published:** 2024-10-10

**Authors:** Mehmet Ozgeyik, Ibrahim Etem Dural, Erkan Baysal, Anthony Li, Basar Candemir

**Affiliations:** ^1^ Department of Cardiology, Cardiovascular Sciences Research Centre, St George’s University of London, London, United Kingdom; ^2^ Department of Cardiology, Afyonkarahisar University of Health Sciences, Afyonkarahisar, Türkiye; ^3^ Department of Cardiology, Diyarbakır Gazi Yasargil Education and Research Hospital, Diyarbakir, Türkiye; ^4^ Department of Cardiology, Ankara University Faculty of Medicine, Ankara, Türkiye

**Keywords:** alcohol ablation, atrial fibrillation, atrial tachycardia, electroanatomic mapping, vein of Marshall (VOM)

## Abstract

Electroanatomic mapping guides complex atrial tachycardia ablations; however, challenges may emerge after pulmonary vein isolation. 3D mapping systems can reveal the mechanism of tachycardia and critical areas that need to be ablated. Sometimes, however, these areas may be located deep inside, to the extent that they cannot be successfully reached by endocardial ablation. In this study, we present a unique case of a patient in whom vein of Marshall (VOM) ethanol ablation, a conventional secondary intervention, promptly terminated a Marshall bundle–related atrial tachycardia without further endocardial radiofrequency application, suggesting VOM ethanol ablation as a potential primary strategy.

## Introduction

Electroanatomic mapping plays a pivotal role in facilitating ablation procedures for complex atrial tachycardias (ATs). However, prompt understanding of the tachycardia mechanism remains crucial for achieving successful ablations. After pulmonary vein isolation (PVI) ablation, approximately 20% of unexpected left-sided atrial tachycardias may manifest, involving epicardial connections that cannot be effectively ablated from the endocardial side (1). The vein of Marshall (VOM) represents a branch within the coronary sinus (CS) venous system, situated in the epicardial aspect of the mitral annulus, typically terminating between the left atrial appendage (LAA) and the left superior pulmonary vein (LSPV) (2). The ablation strategy targets the Marshall bundles (MBs) located at the end of the VOM, which may be associated with tachycardia. When endocardial ablation fails for these ATs, alcohol ablation of the VOM is often utilized as a complementary strategy. In addition, Vlachos et al. showed that ethanol ablation may be related to a better long-term outcome (1). In this report, we present a case of a patient in whom initial VOM alcohol ablation promptly terminated an epicardial AT, rendering it non-inducible without the need for subsequent endocardial radiofrequency application.

## Patient information

A 49-year-old male patient was admitted to our center with a palpitation of 3 months’ duration. He had no other cardiac symptoms. His heart rate was 115 beats/min, blood pressure was 105/72 mmHg, and oxygen saturation rate was 97% in the air room. He was a non-smoker and had neither comorbidities nor structural heart disease predisposing to the arrhythmia. He had undergone three previous left atrial ablations, namely PVI, posterior wall box isolation, and cavotricuspid isthmus ablation. Upon admission, the patient was prescribed rivaroxaban, amiodarone, metoprolol, and ramipril to manage his high heart rate. A full timeline of the patient's history is given in Table 1.

**Table 1:** A timeline of the patient's history.

Date	Patient's history
March 2019	Paroxysmal atrial fibrillation was started when high-frequency episodes of arrhythmia occurred
September 2020	Pulmonary vein isolation with radiofrequency ablation
February 2021	Onset of persistent atrial fibrillation
March 2021	Failed electrical cardioversion in three attempts
August 2021	Redo pulmonary vein isolation (there were electrical connections in the right superior pulmonary vein)
	Posterior box isolation
	Anterior line ablation (between the right superior pulmonary vein and the mitral annulus)
	Cavotricuspid isthmus ablation
September 2022	Onset of drug-resistant persistent atrial tachycardia
**December 2022**	**Vein of Marshall alcohol ablation**
July 2023	6-month follow-up without any tachycardia episode

Bold text is the last and most curative procedure for this patient.

## Diagnostic assessment

**Figure 1:**
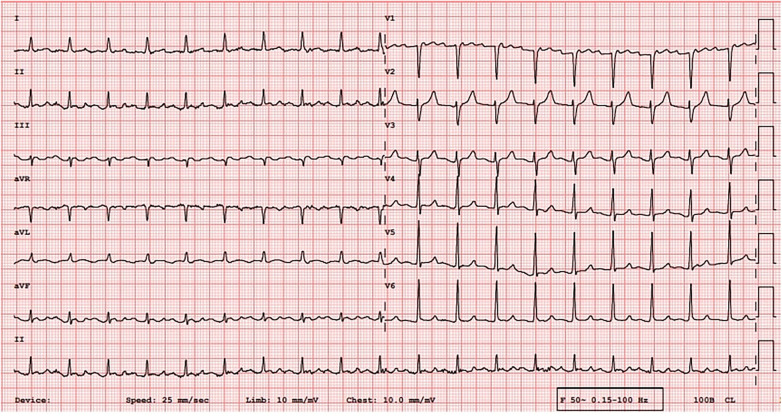
An ECG of persistent left-sided atrial tachycardia at admission.

**Figure 2:**
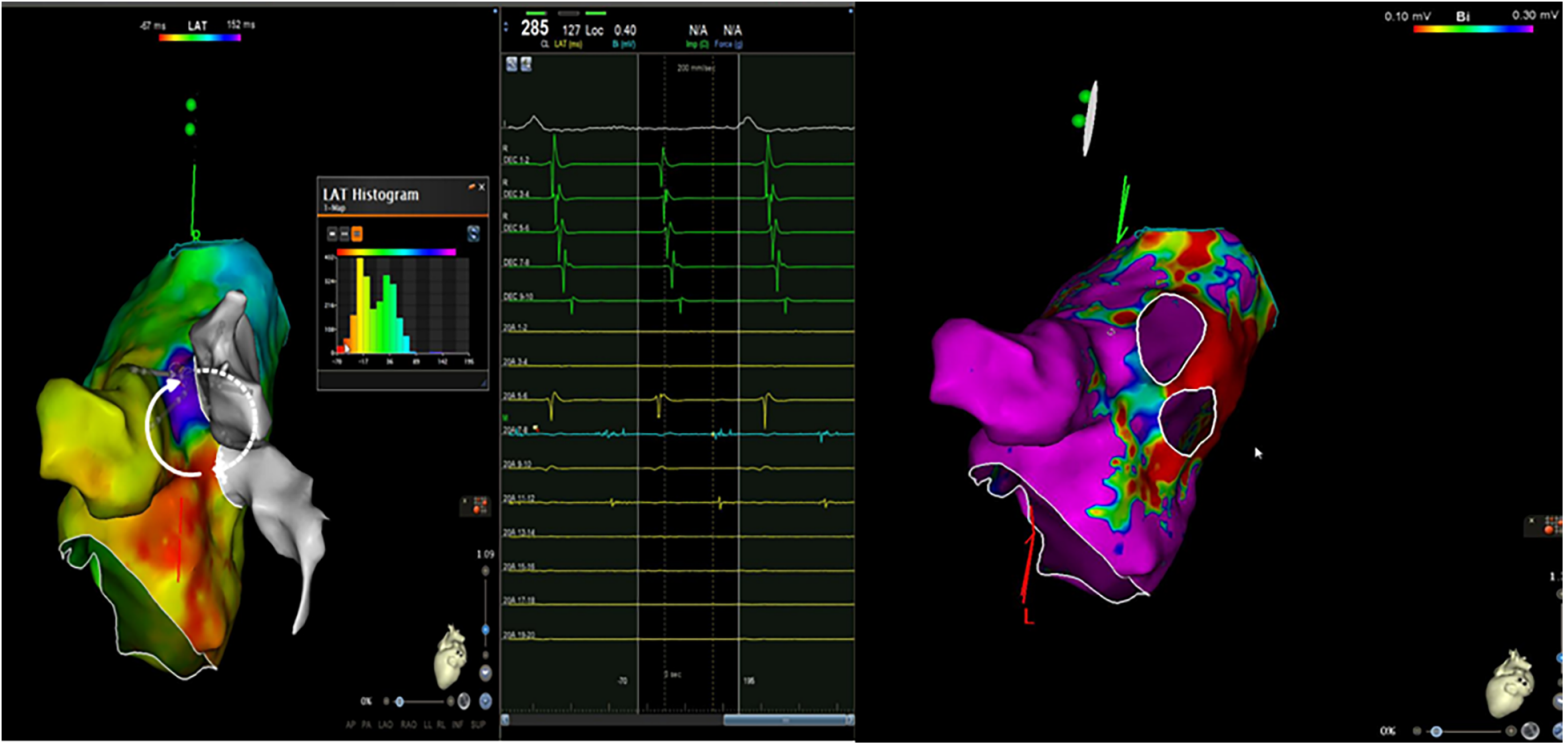
An electroanatomic mapping of the left atria and histogram of the LAT mapping during a tachycardia event and a bipolar map of the left atrium before ethanol ablation of the VOM (the propagation of the local reentry of the tachycardia is shown in the form of white arrows. The white arrows with dots show epicardial activation through the Marshall bundle).

An electrocardiogram (ECG) was quickly performed and an atrial tachycardia was observed (Figure 1). All blood test results were in normal range and there was no sign of infection. Then, echocardiography was performed and the left ventricle's ejection fraction was measured at 52% with normal cardiac structures. Next, an electrophysiological study (EPS) was conducted. The tachycardia cycle length (TCL) of the AT was found to be 285 ms, and CS activation was eccentric and compatible with left atrial origin. The LA was electroanatomically mapped using a CARTO 3D mapping system (Biosense Webster, Diamond Bar, CA, USA) with a high-density mapping catheter (Pentaray). The local activation time (LAT) map demonstrated that all pulmonary veins and the posterior wall were already isolated. A local reentry was detected between the LSPV and the LAA, and wavefront collision was seen on the anterior wall consequent to activation mapping, and therefore, no perimitral AT was suspected. This could have been easily excluded by performing consecutive entraining maneuvers at the CS proximal and distal regions, and these should be done when the mechanism of tachycardia cannot be understood. However, these maneuvers were not performed in this patient because of the fear of unwanted termination of the tachycardia and because the activation map clearly showed no perimitral AT. This area also harbored low-amplitude fragmented diastolic electrograms (EGMs) compatible with the critical region. However, 17% of the tachycardia circuit was found to be missing in the LAT histogram, suggesting another yet-unmapped site, i.e., the epicardial surface (Figure 2). Given the typical proximity of the Marshall bundle to this area, an element of suspicion arose regarding the direct participation of the MB in the tachycardia circuit.

## Therapeutic intervention

**Figure 3:**
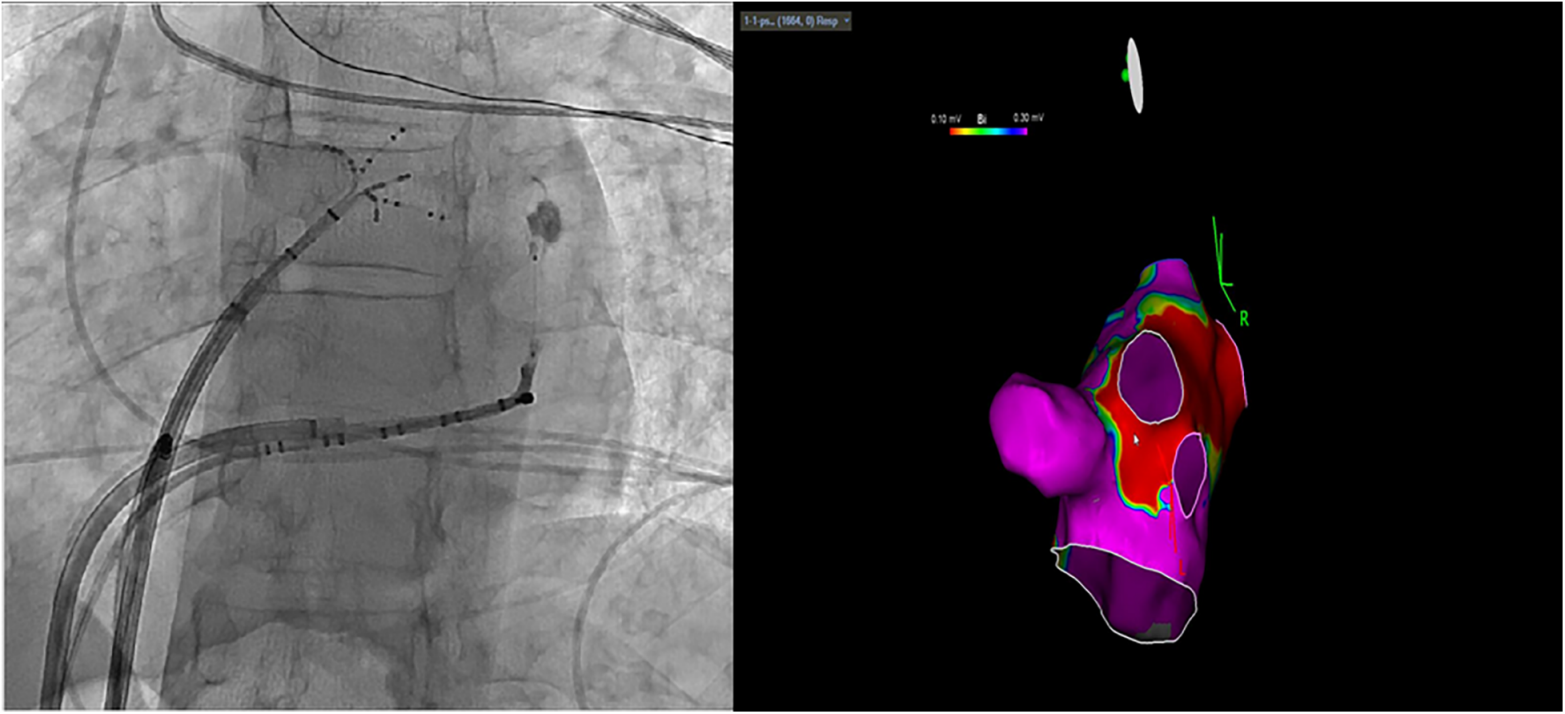
Vein of Marshall venography to demonstrate contrast staining of the affected myocardium and a bipolar map of the left atrium after ethanol ablation of the VOM.

Ethanol ablation for the VOM is typically performed after the failure of endocardial radiofrequency (RF) ablation as a complementary approach in persistent perimitral atrial tachycardias and not as a first option (3–5). But because we suspected direct involvement of the epicardial tissues in the AT mechanism, we opted for ethanol ablation of the VOM as the initial intervention after direct visualization of the VOM as a suitable target. An over-the-wire balloon (2 mm × 15 mm) was placed at the ostium of the VOM and the administration of 3 cc of ethanol was started. The AT promptly slowed down and terminated during the initial phase of the first ethanol infusion (around 1.5 cc). All planned ethanol infusions were completed. After the injections, we repeated VOM venography to demonstrate contrast staining of the affected myocardium (Figure 3). Following the alcohol infusion, a LA voltage map was re-created, which revealed no voltage over critical endocardial sites (Figure 3). Because no tachycardia could be induced, we did not apply any RF endocardially. In addition, because we already excluded a perimitral AT by activation mapping and therefore determined that the mitral isthmus was not involved in the circuit, we ultimately did not aim to achieve a bidirectional mitral block.

## Follow-up and outcomes

The patient was followed up for 6 months, and during this period, he did not experience any symptoms or tachycardia. At the 3rd and 6th month visits, a 72-hour ambulatory ECG monitoring was performed and no arrhythmia was detected.

## Discussion

Electroanatomic mapping utilizing 3D electroanatomic mapping systems significantly facilitates ablation procedures. However, accurate interpretation and annotation of the correct signals are pivotal for precise cardiac mapping (6). In instances where the LAT histogram fails to encompass the entire tachycardia cycle length, consideration must be given to alternative cardiac chambers potentially associated with the tachycardia, such as the epicardial space.

Prudent interpretation of intracardiac signals holds paramount importance in ablation procedures. Particularly crucial are mid-diastolic atrial signals spanning the entire period for pinpointing areas specifically linked to tachycardia. In the case of the patient in this study, the detection of presystolic and late mid-diastolic low-amplitude long-fragmented EGMs between the LAA and the LSPV was notable. The absence of early diastolic signals on the endocardial map suggested possible tachycardia-related sites within both the myocardium and the epicardium. In addition, the unipolar recording in this region demonstrated an rS pattern, signifying a breakthrough originating from an epicardial site (1). Therefore, considering the presumed critical site proximity to the lateral side of the left atrium, we suspected direct involvement of the MB, an epicardial structure.

Different centers adopt varied VOM ethanol administration techniques. Sang et al. employed a protocol involving two 2 ml ethanol infusions (2). In another study, authors administered 6–10 ml ethanol infusions (7). Our clinical experience suggests that lower ethanol amounts are likely to be associated with recurrences. Thus, despite terminating the tachycardia after a 1.5 cc ethanol infusion in our patient, we administered a 3 cc ethanol infusion each time and repeated it three more times. Margato et al. previously conducted successful ethanol ablation of the VOM in a similar patient case (8). Building on this evidence, we opted for exclusively performing ethanol ablation first, successfully terminating the tachycardia and rendering in non-inducible without additional endocardial radiofrequency application.

Alcohol ablation of the VOM is typically a complementary approach after radiofrequency ablation failure. However, in select patients in whom tachycardia is highly presumed to be MB-related, the aforementioned approach can serve as an initial option avoiding unnecessary subsequent endocardial radiofrequency ablations.

## Take-home messages

Electroanatomic mapping has transformed ablation procedures for complex ATs. In patients in whom endocardial ablation fails, alcohol ablation of the VOM offers a complementary strategy. In this study, we presented a case of a patient in whom initial VOM alcohol ablation promptly terminated an epicardial AT, avoiding the need for further endocardial intervention. This underscores the potential of VOM alcohol ablation as an effective first-line option in select patients, particularly when epicardial involvement is suspected, potentially sparing patients from unnecessary endocardial procedures.

## Data Availability

The original contributions presented in the study are included in the article/Supplementary Material, further inquiries can be directed to the corresponding author.

## Supplementary material

Supplementary Video 1 Propagation mapping of left atrium during tachycardia.

Supplementary Video 2 Termination of tachycardia during alcohol infusion.
